# A rapid influenza diagnostic test based on detection of viral neuraminidase activity

**DOI:** 10.1038/s41598-021-04538-4

**Published:** 2022-01-11

**Authors:** Xuexiang Lin, Xiao-Yu Liu, Bo Zhang, Ai-Qing Qin, Kwok-Min Hui, Kevin Shi, Yang Liu, Don Gabriel, X. James Li

**Affiliations:** 1 Shenzhen Institutes of Advanced Technology, Chinese Academy of Sciences, Shenzhen, 518055 China; 2 University of Chinese Academy of Sciences, Beijing, 100049 China; 3 Cellex Incorporated, 76 TW Alexander Drive, Research Triangle Park, NC 27709 USA; 4 College of Science, Shenyang Agricultural University, Shenyang, 110866 China

**Keywords:** Influenza virus, Assay systems, Immunochemistry

## Abstract

Current methods used for diagnosis of acute infection of pathogens rely on detection of nucleic acids, antigens, or certain classes of antibodies such as IgM. Here we report a virus enzyme assay as an alternative to these methods for detection of acute viral infection. In this method, we used a luciferin derivative as the substrate for detection of the enzyme activity of influenza viral neuraminidase as a means for diagnosis of influenza. The resulting commercial test, the qFLU Dx Test, uses a different supply chain that does not compete with those for the current tests. The assay reagents were formulated as a master mix that accommodated both the neuraminidase and luciferase reactions, thereby enabling rapid and prolonged production of stable light signal in the presence of influenza virus in the sample. The assay was evaluated using depository throat swab specimens. As expected, the assay exhibited similar detection rates for all influenza types and subtypes except for A(H7N9), which exhibited lower detection rate due to lower viral titer in the specimens. When throat swab specimens were diluted with the sample buffer of the test kit and tested with the qFLU Dx Test. The sensitivity and specificity were 82.41% (95% confidence interval: 79.66–85.84%) and 95.39% (95% confidence interval: 94.32–96.46%), respectively, for these diluted specimens in comparison to a real-time polymerase chain reaction assay. The uniqueness of the qFLU Dx Test as an enzymatic assay makes it highly complementary with currently available methods.

## Introduction

The ongoing COVID-19 pandemic reveals several significant challenges to diagnostic assays. Current methods rely on detection of viral nucleic acids, antigens, or specific antibodies, which tend to compete with the same sources of key materials and lead to shortage of supplies. Moreover, the SARS-CoV-2 virus mutates rapidly, which may affect the efficacy of these diagnostic assays. Here we propose an alternative method for detection of acute infection of a pathogen. Many viral pathogens contain unique enzymes, the activities of which may be effective diagnostic markers. Some of these enzymes had been successfully used as therapeutic drug targets, indicating that they should also provide sufficient specificity of diagnosis of infection.

Here we report a biochemiluminescent assay for detection of influenza viral neuraminidase (NA) activity as a means for detection of influenza. Influenza viral neuraminidase is an effective target of a newer generation of influenza therapeutic drugs that inhibit the activity of this enzyme, suggesting that a unique and specific substrate can be designed for use in diagnosis. A substrate derivatized with firefly luciferin improves the sensitivity considerably. Further improvement makes the assay suitable for use in point of care settings.

Like SARS-CoV-2 virus, influenza virus constantly mutates itself, and can easily be transmitted from one individual to another, annually affecting 5–10% adults and 20–30% children in the world^1,2^. It is estimated that globally, annual epidemics of influenza lead to 3–5 million cases of severe illness, which may require hospitalization, and about 250,000–500,000 deaths^3^. An uncontained pandemic influenza may lead to far worse consequences^4^. Rapid diagnosis of epidemic or pandemic influenza plays an important role in effective management of influenza and public health.

Rapid influenza diagnosis tests (RIDTs) intended for detection of active infection, which can be completed within 30 min, fall into two categories, i.e., those that detect viral antigens and those that detect viral nucleic acids. The antigen assays have a number of drawbacks, the most significant one being lack of sensitivity^4–8^. Molecular-based influenza assays, which detect the viral nucleic acids, offer better sensitivity. Among these tests, however, only a few can be completed within 30 min^9–14^ and thus suitable for point of care use. Antigen and nucleic acid assays suffer from a common drawback in that they all rely on static epitopic or genetic sequences, which are susceptible to mutations. Indeed, the lateral flow based RIDTs were found to be less sensitive for detection of the pH1N1 virus during the H1N1 pandemic^15^.

Influenza viral NA activity as a diagnostic marker for influenza had been previously explored. A colorimetric substrate specific for influenza viral NA was developed and successfully used in an assay for detection of influenza, demonstrating the feasibility of this strategy^16–20^. A more sensitive chemiluminescent substrate derivatized with dioxetane was subsequently developed and used in a test for influenza diagnosis^21,22^. Although the chemiluminescent assay had 88% clinical sensitivity in comparison to culture method when nasal aspirate specimens were used^23,24^, the assay was too complex for it to be widely adopted for clinical use, particularly for point of care use.

**Figure 1:**
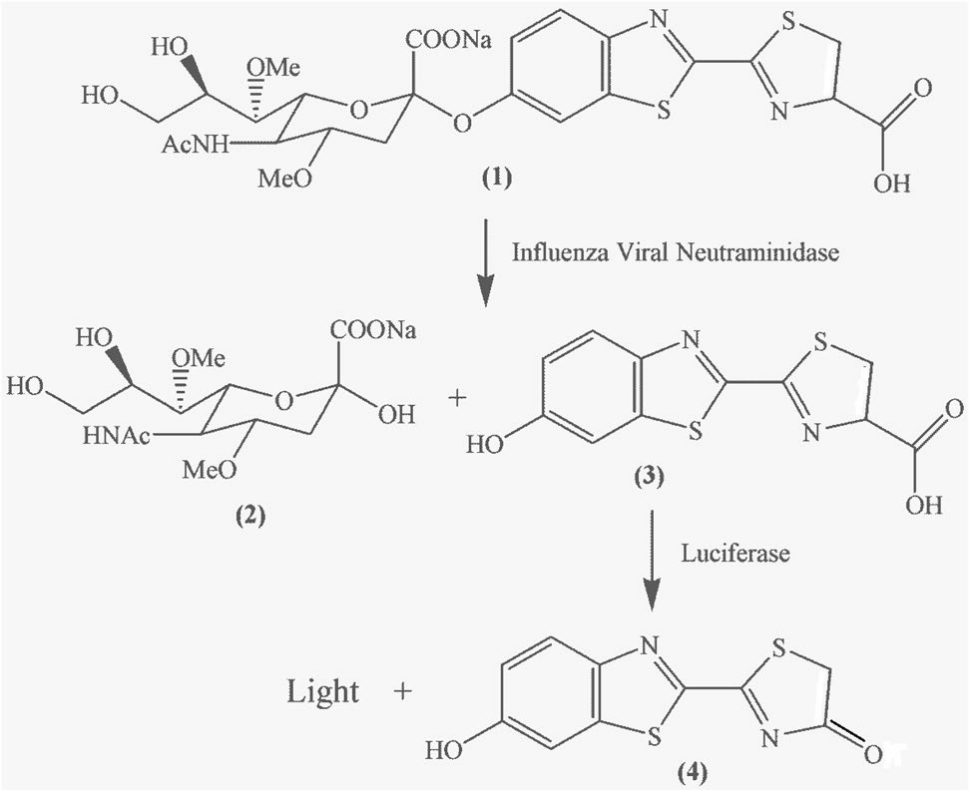
Biochemiluminescence Reaction. The qFLU Dx Test uses 4, 7-dimethyl neuraminic acid -O-luciferin as the substrate for detection of influenza virus. In the presence of Type A or B influenza virus, viral neuraminidase cleaves the substrate to release the luciferin moiety, which is oxidized to oxyluciferin by luciferase to generate light signal. Since the substrate is continuously cleaved the neuraminidase to result in luciferin that is continuously oxidized to generate light signal, this is a “real time” assay with glow light feature.

Here we report a biochemiluminescent assay using a luciferin derivatized substrate for specific detection of influenza viral NA activity^3,25,26^. The assay reagents were formulated as a lyophilized master mix to simplify the assay procedure, which essentially is a one-step assay (Fig. 1). Presence of influenza viral NA in the reaction generates stable light signal, which lasts for at least 2 h. The stable light signal can be detected using a simple luminometer. The entire detection procedure takes approximately 15 min. Because firefly luciferase/luciferin is a highly sensitive biochemiluminescence, the qFLU Dx Test is sensitive. The present work demonstrated the feasibility of using viral enzyme activity as a means for detection of acute viral infection.

## Materials and methods

### qFLU Dx test procedure

The assay is referred to as the qFLU Dx Test or Assay. All reagents were present in a master mix that was lyophilized as a reagent bead. One reagent bead is for one reaction. The reaction is initiated upon addition of a sample in the sample buffer. The resulting reaction solution contains the following active ingredients: 0.012 mg/mL substrate, 0.024 mg/mL firefly luciferase, 2 mM ATP, 10 mM HEPES (pH 7.2), 15 mM MgCl_2_, and 4 mM CaCl_2_. The substrate and luciferase were produced in-house by Cellex while the other ingredients were purchased from Sigma or FishserScientific.

As illustrated in Fig. 1, the substrate is cleaved by the NA in a sample to release luciferin, which is then detected with luciferase that is present in the master reagent mix. Presence of influenza viral NA in the reaction generates stable glow light signal that can be detected with a luminometer.

A swab sample was inserted into qSample buffer tube (2.0 mL) for sample elution. To perform detection, 0.25 mL of the sample was added to the test tube containing a master mix reagent bead. Following swirling of the sample solution to dissolve and mix the reagent bead, which took approximately 10 s, the reaction tube was incubated at room temperature for 15 min. The test tube was then placed in Helios 2000 Version 1.0 Analyzer for signal detection. Cutoff value in relative light unit (RLU) was pre-set for the Helios 2000 analyzer.

For samples that were not collected in qSample Buffer, the samples were diluted 1:1 with 2× qSample Buffer and then used for detection as described above.

Signal to cutoff value (S/CO) was used to interpret the test results. Since the relative light units (RLU) were used for luminometers and different brand of luminometers may define RLU differently, luminometers from different manufacturers should be calibrated. The cutoff value (CO) used in this study was set at 220,000 for Helios 2000 luminometer by testing approximately 400 negative nasal swab specimens. Helios 2000 was then used to calibrate the RLU of Helios 200 by repeatedly testing serially diluted samples. The cutoff RLU value for the Helios 200 was 220 (1/1000 of the Helios 2000 cutoff value). Samples with a S/CO value of 1.0 or above were considered positive.

### Sources and propagation of microbial organisms

Influenza virus strains A/CA/072009 and A/NC/39/2009 were from Dr. Larisa Gubareva’s laboratory of the Centers for Disease Control and Prevention (CDC). These virus strains were propagated in Madin–Darby Canine Kidney (MDCK) cells according to the WHO protocol. Other influenza viruses were purchased from ATCC and used without propagation. The subtypes were provided for all type A virus (Table 2). The type B virus isolates tested were collected before 1983 when there was only one lineage of circulating type B virus.

### Detection of retrospectively collected clinical specimens

These specimens were collected during 2011/2012 and 2012/2013 influenza seasons in Asia as part of a public health surveillance program. The clinical specimens were collected as throat swabs, eluted in 3 mL of veal infusion broth or Hank’s salt solution and frozen at − 70 °C or below. 0.4 mL of the sample was subjected to nucleic acid extraction using magnetic particles. The resulting nucleic acids were used in a real time polymerase chain reaction (PCR) assay for detection and typing of influenza viruses using the AgPath-ID™ One-Step RT-PCR Kit (Life Technologies, CA) and primers and probes recommended by World Health Organization (WHO). 0.3 mL of the sample was diluted with an equal volume of 2× qSample Buffer. 0.25 mL of the diluted sample was used for the qFLU Dx Test. Since these samples were not collected specifically for the present study and were unlinked from the donor identities, the study described here was not considered one, which involved human subjects.

### Data analyses

Excel software was used for statistics analyses.

### Instruments

qFLU Dx Test uses a luminometer for detection of light signal. Portable desktop luminometers Helios 200 and Helios 2000 were used to measure the light signal in the studies.

## Results

### Reaction kinetics

**Figure 2:**
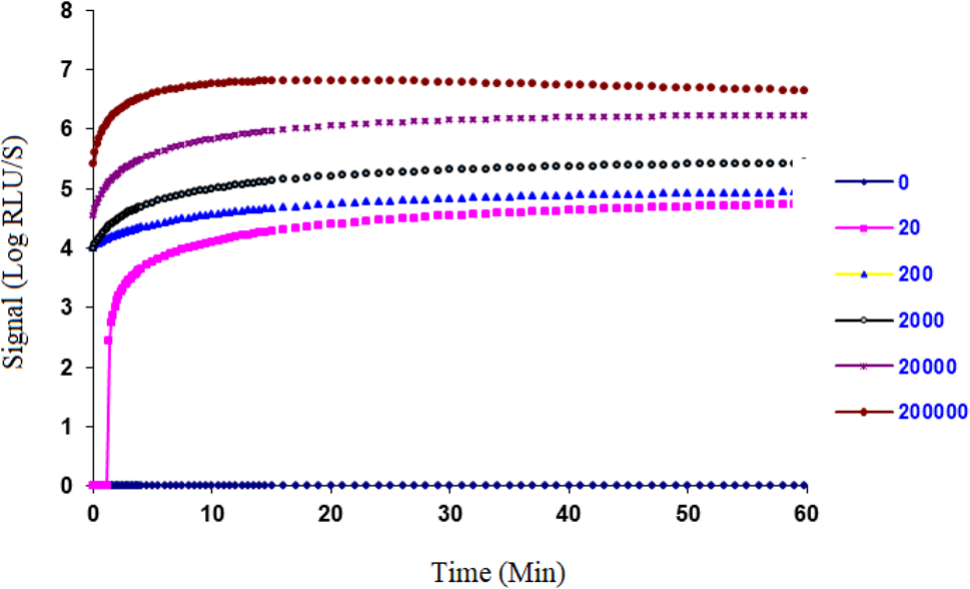
Reaction kinetics. A reaction was initiated by adding 250 µL of sample containing an influenza virus to qFLU Dx Test master mix bead. The sample contained various concentration of virus (0–200,000 TCID50/mL. Compared to the negative sample, positive signal was detected within 2 min and stabilized at about 5 min. The signal was collected over a period of 60 min for each reaction. The signal intensity (RLU) was plotted against the time to establish a time course reaction kinetics.

To assess the chemiluminescence reaction kinetics of the qFLU assay, a cultured influenza virus strain (A/WS/33) was diluted with 1× qSample Buffer to various concentrations and tested using the qFLU Dx Test. Signal intensity was recorded over a period of 60 min. The data showed that the signal reached plateau at approximately 5 min and was stable for at least 60 min after the initiation of reactions (Fig. 2). Thus, valid signal could be collected for influenza diagnosis between 5 and 60 min. A 15 min incubation at room temperature before signal detection was used for the qFLU Dx Test during the current studies.

### Assay linear range

**Figure 3:**
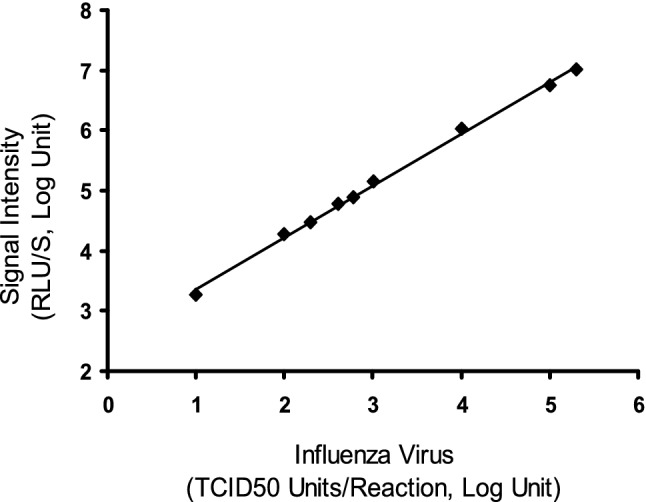
Linearity and linear range. A scattering plot between the signal intensity (relative light units, RLU) collected at 15 min after reaction initiation and influenza virus concentrations of A/pH1N1/CA/07/2009, both in log units. There was a linear relationship (R2 > 0.99) between the signal intensity (RLU) in log units and virus concentrations in log units with a linear range of greater than 4 logs.

The qFLU Dx Test was designed to be a semi-quantitative assay. This study was carried out to assess the analytic linear range of the qFLU Dx Test. Samples containing various concentrations of an influenza virus strain (A/CA/07/2009) were tested with the qFLU Dx Test. A scattering plot using the log transformed virus concentrations and signal intensity (RLU) was constructed to estimate the correlation coefficient (R2) over the virus concentration range. The correlation coefficient (R2) was 0.9967 (95% CI 0.9690–1.0; Fig. 3) over a range of 4 logs.

### Assay analytical sensitivity

To assess the analytical sensitivity of the qFLU Dx Test, a recombinant NA derived from A/turkey/Wisconsin/1/1966 (H9N2) and produced in Sf9 insect cells as a His-tagged protein was sequentially diluted in 1× qSample Buffer. Each diluted sample was tested in five replicates. Quantitative signal intensity related to neuraminidase concentrations was observed (Table 1). Signals above the background were detected when NA concentration was 0.08 ng/mL or higher; above cutoff signals (220,000) were detected when NA concentration was 2.22 ng/mL or higher (Table 1).

**Table 1:** A recombinant influenza viral neuraminidase was serially diluted in qSample buffer.

Sample no	1	2	3	4	5	6	7	8
NA (ng/mL)	60.00	20	6.67	2.22	0.74	0.25	0.08	0.03
Mean RLU (n = 5)	5476.0	1978.8	692.8	255.2	110.2	42.4	26.6	− 6.4
Standard deviation	377.82	60.43	17.08	15.24	6.69	4.04	3.91	9.53
Coefficient of variation (%)	6.90	3.05	2.47	5.97	6.07	9.52	14.70	N/A

Five (5) replicates were tested for each concentration. The mean signal intensity (RLU) was the average signal minus the dark counts (background count). Standard deviation (SD) and coefficient of variation (CV) were computed using the Excel software.

### Limit of detection

Limit of detection (LoD) was defined as the lowest concentration tested at which 95% of replicates was positive but further dilution would result in less than 95% positivity rate. The virus stock was first serially diluted, and three replicates were tested. A concentration resulting in all three positive test results but one more dilution resulting in at least one or more negative test results was considered preliminary LoD concentration. Twenty replicates were then tested at the preliminary LoD concentration. If at least 19 of the twenty replicates were tested positive, the concentration was considered the LoD concentration. Two influenza virus strains (A/pH1N1/CA/07/2009 and A/pH1N1/NC/37/2009) were diluted to concentrations approaching the limits of detection with signal to cutoff (S/CO) ratios close to 1.0. Twenty replicates were tested for each dilution for both virus strains. 100% replicates were positive for strain A/CA/07/2009 when tested at 995 TCID_50_/mL or higher and for strain A/NC/37/2009 when tested at 953 TCID_50_/mL or higher (Table 2). Thus, the LoD for A/pH1N1/CA/07/2009 and A/pH1N1/NC/37/2009 were 995 and 953 TCID_50_/mL, respectively.

**Table 2:** Various virus strains were diluted to concentrations that generated a signal intensity close to the cutoff signal.

Virus Strain	Replicates (n)	TCID_50_/mL or CEID_50_/mL	Signal (RLU × 1000)	Mean S/CO
A/pH1N1/CA/07/2009	20	995	347	1.58
A/pH1N1/NC/39/2009	20	953	253	1.15
A/H1N1/PR/8/34	3	0.8	284	1.29
A/H1N1/FM/1/47	3	0.07	283	1.29
A/H1N1/NWS/33	3	5330	244	1.11
A/H1N1/Denver/1/57	3	53,300	270	1.23
A/H1N1/New Jersey/8/76	3	741	284	1.29
A/H3N2/Port Chalmers/1/73	3	6846	265	1.20
A/H3N2/Hong Kong/8/68	3	2330	384	1.75
A/H3N2/Aichi/2/68	3	13	282	1.28
A/H3N2/Victoria/3/75	3	158	290	1.31
B/Lee/40	3	2.50	287	1.30
B/Allen/45	3	1.98	280	1.28
B/GL/1739/54	3	0.11	285	1.30
B/Taiwan/2/62	3	8.90	259	1.18
B/Hong Kong/5/72	3	528	313	1.42
B/Maryland/1/59	3	1.48	305	1.39

Strains A/pH1N1/CA/07/2009 and A/pH1N1/NC/2009 were from CDC, propagated, and tittered before testing. Twenty replicates of A/pH1N1/CA/07/2009 and A/pH1N1/NC/2009 were tested at 995 and 993 TCID50/m, respectively; all replicates tested positive at these virus concentrations. Other strains were purchased from ATCC, diluted to concentrations near the cutoff and tested without further propagation. Note that the virus concentrations near the cutoff value varied considerable among different strains of virus. S/CO: signal to cutoff ratio.

Other influenza virus strains were purchased from ATCC, diluted in 1× qSample Buffer without propagation and tested in triplicate at various concentrations. All influenza virus strains exhibited NA activity and could be detected with the qFLU Dx test. The lowest concentration resulting in positive detection for all replicates is listed in Table 2 for all virus strains.

The virus isolates used in this study were collected from 1933 to 2009. All showed neuraminidase activity that could be detected with the qFLU Dx Test, indicating that despite expected mutations accumulated over time, these viruses have retained the neuraminidase activity detectable with the substrate used in the qFLU assay.

### Detection of retrospectively collected clinical specimens

The qFLU Dx Test was evaluated using 2215 frozen clinical specimens collected as throat swabs during 2011/2012 and 2012/2013 influenza seasons. All these specimens had been previously tested and, if positive, typed/subtyped with RT-PCR assay according to the WHO protocol (for an updated protocol, please refer to https://www.who.int/influenza/gisrs_laboratory/Protocols_influenza_virus_detection_Jan_2020.pdf). Since these specimens were not eluted in qSample Buffer, 0.3 mL of the specimens were mixed with an equal volume of 2× qSample Buffer and tested with the qFLU Dx Test. The PCR test results were unknown to the technicians before the qFLU Dx Test was performed.

Of the 2215 specimens, there were 739 RT-PCR-positive specimens, of which there were 593 and 146 influenza virus Types A and B, respectively. Of the 593 influenza virus Type A specimens, there were 173 A(H1N1), 95 A(pH1N1), 305 A(H3N2), and 20 A(H7N9) specimens.

The RT-PCR method was used as a comparator test for estimation of the sensitivity and specificity of the qFLU Dx Test. Overall, the qFLU Dx Test had a sensitivity and specificity of 82.41% (95% confidence interval: 79.66–85.84%) and 95.39% (95% confidence interval: 94.32–96.46%), respectively, when compared to the RT-PCR assay (Table 3). Both types A and B virus had similar sensitivity of 82.80% and 80.82%, respectively. In addition, all influenza virus A subtypes except for A(H7N9) had similar sensitivity (Table 4). The qFLU Dx Test detected 14 of the 20 RT-PCR positive A(H7N9) specimens. It is interesting to note that the average signal strength (RLU) for the specimens containing the H7N9 virus was significantly lower than the other virus subtypes and Type B virus (Table 4). This may be due to lower viral load, lower specific activity of the enzyme, fewer enzyme molecules per virion particle, or other unknown reasons.

**Table 3:** The sensitivity and specificity of the qFLU Dx Test were computed using the RT-PCR test results as the comparator test.

		qFLU Dx test		
		Positive	Negative	Subtotal
Real time PCR	Positive	609	130	739
	Negative	68	1408	1476
	Subtotal	677	1538	2215
Sensitivity	82.41%	95% CI 79.66–85.84%		
Specificity	95.39%	95% CI 94.32–96.46%		

*The 95% CI* 95% confidence interval.

**Table 4:** The RT-PCR positive samples were grouped according to influenza virus types (A or B) and Type A subtypes (H1N1, pH1N1, H3N2 and H7N9).

Type	Subtype	PCR positive (n)	qFLU Dx test		
			Detected positive (n)	Detection rate (%)	Average signal (RLU × 1000)
B	N/A	146	118	80.82	1215
A	H1N1	173	142	82.08	3073
	pH1N1	95	78	82.11	2131
	H3N2	305	257	84.26	3016
	H7N9	20	14	70.00	356

The detection rates of the qFLU Dx Test were calculated for Type B and Type A subtypes. Note that the signal intensity for the H7N9 specimens was considerably lower than the other types or subtypes.

The throat samples used in the study were collected in 3 mL virus transport medium, which was further diluted 1:2 or 6 mL total volume/swab for use in qFLU Dx Test. In contrast, an antigen test normally uses a nasal swab eluted in 1 mL the swab specimen or about 6 times as concentrated as the specimens used in the present study.

## Discussion

Diagnostic assays, particularly those that can be used in point of care settings, play an important role in pandemic and epidemic management as vividly demonstrated in the ongoing COVID-19 pandemic. Based on the markers they detected, these diagnostic assays fall into two broad categories, the molecular assays which detect nucleic acids and immunoassays that detect antibodies or antigens. Here we reported another type of assay, which detects viral enzyme activities for detection of a viral infection.

Most pathogenic viruses contain unique enzymes, which can be used as diagnostic markers. Viral enzyme assays provide several unique features. The key materials used in these assays are different from those used in molecular assays or immunoassays. Consequently, the enzyme assays do not compete with the supply chains of molecular assays or immunoassays, which is important as a pandemic may cause sudden and huge increase in demand of diagnostic tests. In addition, enzyme assays are generally less susceptible to virus mutations, which can happen during a pandemic or epidemic such as the ongoing COVID-19 pandemic. The present study showed that the influenza virus isolates collected in 1930’s could still be detected with the qFLU Dx Test for their neuraminidase activity (Table 1). On the other hand, molecular assays and immunoassays are vulnerable to virus mutation as they depend on nucleic acid or protein sequences. Moreover, the enzyme assays can also be converted to drug resistance tests as many of these enzymes are also pharmaceutical drug targets.

The qFLU Dx Test described here is a biochemiluminescence assay, which detects the enzyme activity of influenza viral neuraminidase. In the presence of influenza virus, the assay produced stable light signal, which could be detected with a simple luminometer. The assay was simple and rapid. When throat swab specimens were used and tested in 1:2 dilution (one part specimen to two parts of qSample buffer), the assay showed an overall sensitivity and specificity of 82.41% and 95.39%, respectively (Table 3). The assay showed similar detection rates for all influenza virus types/subtypes except for A (H7N9). The lower detection rate for A/H7N9 was likely due to lower viral load in the specimens, lower specific activity of the neuraminidase of this subtype virus, fewer NA molecules per virion particle, or other unknown reason (Table 4). Since neuraminidase activity of influenza virus is involved in dissimilation of the virus, the lower signal in H7N9 clinical specimens is worthy further investigation.

The assay showed remarkable analytical sensitivity at less than 1 ng/mL neuraminidase. The clinical sensitivity was lower than expected. The lower clinical sensitivity can be attributed to the use of throat swab specimens, which were eluted in a large volume and were further diluted in the present study. Use of nasal swab specimens eluted in smaller volume of buffer without dilution should result in much improved clinical sensitivity as nasal swabs contain much higher viral load and sample dilution can lower the sensitivity.

As expected, the substrate used in the qFLU Dx Test showed high specificity in analytical studies without cross reactivity with other viruses or bacterial species (see Supplemental Data). The test can tolerate a wide range of potentially interfering substances (Supplemental Data). These performance characteristics suggest that the qFLU Dx Test is suitable for clinical use.

In summary, the data presented here showed that the qFLU Dx Test, which is based on detection of influenza viral enzyme for influenza diagnosis, may be a viable alternative for rapid diagnosis of influenza. This assay is easy to use, rapid and, thus, suitable for point of care use. Many pathogenic viruses also have enzymes that can be exploited for use as diagnostic markers.

## Supplementary Information

Supplementary Information.

## Data Availability

The data used to support the results of this work are available from the corresponding author upon request.
