# Supplementary material for: A rapid influenza diagnostic test based on detection of viral neuraminidase activity
Source: Sci Rep. 2022 Jan 11;12:505. doi: 10.1038/s41598-021-04538-4 (PMC8752744; doi:10.1038/s41598-021-04538-4)
Supplement: Supplementary file 1 — Supplementary Information. [file 41598_2021_4538_MOESM1_ESM.docx]

SUPPLEMENTAL DATA

**Precision**

The precision study was designed to evaluate assay variability. Two sub-studies were conducted. The first study was performed in three sites to evaluate site-to-site reproducibility. The second study was performed in one site over a period of 12 days to assess within-site repeatability.

The study used a sample panel consisting of an OC resistant virus (samples #1-3), an OC susceptible H3N2 virus (samples #4 and 5) and a negative sample (the Q-Sample buffer; #6). The influenza positive samples were diluted to low to medium titers. Except for the negative control, all samples were prepared in aliquots, frozen and shipped to the study sites. The negative control (1X Q-Sample buffer) was prepared in a bottle and stored at room temperature at the study sites.

**a. Site-to-Site Reproducibility**

Three study sites and two operators in each site were involved in the study. Each operator tested three replicates each day over a period of five days. A total of 90 replicates were tested for each sample. Presence or absence of influenza virus was correctly determined for all samples and replicates **(Table 1)**. The coefficients of variation for all influenza virus positive samples were less than 21% except for the negative samples **(Table 1)**. As expected, higher variability was observed for those samples with lower RLU such as the negative sample. These data demonstrated that the QFLU Test is highly reproducible from site to site.

**Table 1 │Site-to-Site Reproducibility**

|  | Positive Sample | | | Negative Control |
| --- | --- | --- | --- | --- |
|  | 1 | 2 | 3 |  |
| Replicates | 90 | 90 | 90 | 90 |
| Mean (RLU) | 371 | 1276 | 5402 | 58 |
| SD | 78 | 197 | 1006 | 45 |
| %CV | 20.99 | 15.26 | 18.63 | 78.88 |
| % Positive Detection Rate | 100 | 100 | 100 | **0** |

**b. Within Site Repeatability**

The same study panel used for the Site-to-Site Reproducibility Study was used for this study. The samples were tested over a period of 12 days in one site. Two runs and two replicates per run were performed daily. As shown in Table 2, presence or absence of influenza virus was correctly determined for 100% of the replicates for all samples (95% CI: 92.75% - 99.95%).

**Table 2 │Within Site Repeatability**

|  | Positive Sample | | | Negative Control |
| --- | --- | --- | --- | --- |
|  | 1 | 2 | 3 |  |
| Replicates | 48 | 48 | 48 | 48 |
| Mean (RLU) | 373 | 1009 | 3933 | 101 |
| SD | 82 | 198 | 1003 | 34 |
| %CV | 21.91 | 19.68 | 25.50 | 33.59 |
| % Positive Detection Rate | 100 | 100 | 100 | 0 |

**Analytical Specificity (Cross Reactivity)**

This study was designed to ensure that substances and microbes, which could be found in samples from patients with flu-like symptoms, would not cause false positive or negative. When tested at a concentration within the linear range, the qFLU Dx Test can detect significant inhibition or enhancement of the signal. Thus, a sample containing a mid-concentration of the wild type virus A/CA/07/2009 was spiked with a substance or microbe and tested with the qFLU Dx Test. An interfering substance or microbe is defined as one, which causes increase or decrease in signal by more than 20% when compared to the control.

**a. Potentially Interfering Substances**

The sample spiked with a substance at an indicated concentration was tested in triplicate with the qFLU Dx Test. All substances showed less than 20% changes in signal when compared to the control, indicating that these substances had no significant impact on the assay when present at or below the listed concentrations **(Table 3)**.

**Table 3 │**Potentially Interfering Substances

| Item # | Substance | Tested Concentration | Signal (RLU) | % Change |
| --- | --- | --- | --- | --- |
| 0 | No sub control | N/A | 3,228 | 0 |
| 1 | Whole Blood (EDTA) | 0.25% | 2,956 | -8.44 |
| 2 | Mucin | 0.25% | 2,650 | -17.91 |
| 3 | Phenylephrine | 0.10% | 2,851 | -11.69 |
| 4 | Oxymetazoline | 0.005% | 3,178 | -1.55 |
| 5 | Sodium Chloride with preservative | 10% | 2,889 | -10.50 |
| 6 | Dexamethasome | 0.5 mg/mL | 3,225 | -0.08 |
| 7 | Beclomethasome | 0.5 mg/mL | 3,263 | 1.08 |
| 8 | Flunisolide | 0.5 mg/mL | 3,201 | -0.84 |
| 9 | Triamcinolone | 0.5 mg/mL | 3,195 | -1.02 |
| 10 | Fluticasone | 0.5 mg/mL | 3,172 | -1.72 |
| 11 | Menthol | 0.5 mg/mL | 2,779 | -13.92 |
| 12 | Tobramycin | 0.5 mg/mL | 3,254 | 0.81 |
| 13 | Nasal Gel | 10% | 3,114 | -3.52 |
| 14 | Benzocaine | 0.05 mg/mL | 2,835 | -12.19 |

**b. Non-Influenza Human Viruses**

Twelve non-influenza human viruses were procured from ATCC and tested in triplicate at 1:10 dilution. The resulting concentrations are listed in **Table 4**. When compared to the control, all but three viruses showed no significant impact on qFLU Dx Test.

**Table 4 │**Potentially Interfering Viruses

| Item # | Virus | Concentration (TCID_50_/mL or EID_50_/mL) | Signal (RLU) | % Change |
| --- | --- | --- | --- | --- |
| 0 | No virus control | N/A | 2,859 | 0 |
| 1 | Human Adenovirus Type 1 | 5x10^5.5 | 2,535 | -11 |
| 2 | Human Adenovirus Type 7 | 5x10^4.75 | 2,659 | -7 |
| 3 | Human Coronavirus | 1.6x10^5 | 2,644 | -8 |
| 4 | Human Herpesvirus 4 | 5x10^3.5 | 2,075 | -27 |
| 5 | Human Herpesvirus 5 | 5x10^3.5 | 2,314 | -19 |
| 6 | Human Enterovirus | 1.6x10^7 | 2,613 | -9 |
| 8 | Human Parainfluenza Virus Type 2 | 1x10^7 | 2,797 | -2 |
| 10 | Measles | 3.4x10^3 | 2,741 | -4 |
| 12 | Human Respiratory Syncytial Virus (RSV) | 1:10 dilution | 2,800 | -2 |
| 13 | Rhinovirus | 5x10^5.5 | 2,740 | -4 |

**c. Microbes**

Microbes were spiked into a positive sample at the listed concentrations and tested in triplicate with the qFLU test. Three or four microbe and a control were tested in a group. None of them was found to interfere with the qFLU Dx Test as the presence of none of the microbe in a sample at the tested concentration caused a change of more than 30% **(Table 5)**.

When tested at higher concentration, *i.e*, 10^6^ CFU/mL, Streptococcus pneumoniae resulted in signal increase by more than 30%, indicating that high concentrations of Streptococcus pneumonia in a sample may lead to false positive test results.

**Table 5 │**Cross-Reactivity of Microbes

| Item # | Bacteria |  | Rep 1 | Rep 2 | Rep 3 | Average | % Change |
| --- | --- | --- | --- | --- | --- | --- | --- |
| N/A | No Microbial Control | N/A | 2696 | 2707 | 2806 | 2736 | - |
| 1 | *Chlamydia pneumoniae** | 5x10^4.5 | 2022 | 2080 | 2025 | 2042 | -25.36 |
| 2 | *E coli* | 1x10^6 | 2762 | 2670 | 2681 | 2704 | -1.17 |
| 3 | *Mycoplasma pneumoniae* | 2x10^5 | 2446 | 2507 | 2591 | 2515 | -8.10 |
| 4 | *Streptococcus aureus* | 1x10^6 | 2670 | 2745 | 2696 | 2704 | -1.19 |
| 5 | *Streptococcus epidermidis* | 1x10^6 | 2760 | 2823 | 2812 | 2798 | 2.27 |
|  |  |  |  |  |  |  |  |
| N/A | *No Microbial Control* | N/A | 2527 | 2736 | 2780 | 2681 | - |
| 6 | *Streptococcus pyogenes* | 1.7x10^6 | 2794 | 2762 | 2745 | 2767 | 3.21 |
| 7 | *Haemophilus influenzae* | 1.2x10^6 | 2794 | 2736 | 2780 | 2770 | 3.32 |
| 8 | *Neisseria sp* | 1.5x10^6 | 2687 | 2708 | 2771 | 2722 | 1.53 |
| 9 | *Steptococcus salivarius* | 2.5x10^6 | 2916 | 2655 | 2777 | 2783 | 3.79 |
|  |  |  |  |  |  |  |  |
| N/A | *No Microbial Control* | N/A | 2654 | 2710 | 2808 | 2724 | - |
| 10 | *Neisseria meningitidis* | 3.7x10^6 | 2695 | 2803 | 2916 | 2805 | 2.96 |
| 11 | *Moraxella catarrhalis* | 3.8x10^6 | 2782 | 2930 | 2858 | 2857 | 4.87 |
| 12 | *Streptococcus pneumoniae* | 1.4x10^4 | 2965 | 2962 | 2959 | 2962 | 8.74 |
